# Supplementary material for: Content-rich biological network constructed by mining PubMed abstracts
Source: BMC Bioinformatics. 2004 Oct 8;5:147. doi: 10.1186/1471-2105-5-147 (PMC528731; doi:10.1186/1471-2105-5-147)
Supplement: Additional File 2 — The original results of the above study (non-essential files are deleted to keep the file size under the limit set by BMC bioinformatics). [file 1471-2105-5-147-S2.bz2 › chilibotAdditionalFile2/dip20/201ID8652557E690/html/ERCC6_ERCC5.html]

 


 **ERCC6** and **ERCC5** 
  
Found 21 abstracts in PubMed, retrieved 20.  
 

 What does Google say? 
 PDF only 
| .edu only 

---

**Interactive relationship** (e.g. stimulation, inhibition, etc)

**Neutral relationship**- Northern blot analysis revealed that XPG  [ **ERCC5** ]  mRNA was more extensively transcribed in CSB  [ **ERCC6** ]  cells treated with HuIFN beta than in those without HuIFN beta treatment.  Ref: 9864391 Int J Mol Med, 1999
- XPG  [ **ERCC5** ]  protein interacts with multiple subunits of TFIIH and with CSB  [ **ERCC6** ]  protein.  Ref: 8652557 Biochemistry, 1996
- Interactions involving the human RNA polymerase II transcription nucleotide excision repair complex TFIIH, the nucleotide excision repair protein XPG  [ **ERCC5** ] , and Cockayne syndrome group B CSB  [ **ERCC6** ]  protein.  Ref: 8652557 Biochemistry, 1996
- Here, we provide evidence that CSB  [ **ERCC6** ]  is found not only in the nucleoplasm but also in the nucleolus within a complex CSB  [ **ERCC6** ]  IP 150 that contains RNA pol I, TFIIH, and XPG  [ **ERCC5** ]  and promotes efficient rRNA synthesis.  Ref: 12419226 Mol Cell, 2002

**Non-interactive relationship** (e.g. studied together, co-existance, homology, etc.)

- CONCLUSIONS Reduced expression of ERCC1, XPB ERCC3, XPG  [ **ERCC5** ]   **ERCC5** , and CSB  [ **ERCC6** ]   **ERCC6**  is associated with a more than two fold increased risk of SCCHN.  Ref: 11900225 Cancer, 2002
- In contrast to cells bearing mutations in other components of the nucleotide excision repair apparatus XPB, XPD, XPG  [ **ERCC5** ]  and CSB  [ **ERCC6** ] , cells defective for the ERCC1 XPF structure specific nuclease are highly sensitive to cisplatin.  Ref: 12202770 Nucleic Acids Res, 2002
- In a pilot case control study, we measured the relative expression levels of five NER genes ERCC1, XPB ERCC3, XPG  [ **ERCC5** ]   **ERCC5** , CSB  [ **ERCC6** ]   **ERCC6**  and XPC ERCC, excision repair cross complementing.  Ref: 10910954 Carcinogenesis, 2000
- RESULTS The levels of ERCC1, XPB ERCC3, XPG  [ **ERCC5** ]   **ERCC5** , and CSB  [ **ERCC6** ]   **ERCC6**  transcripts were lower in cases than in controls.  Ref: 11900225 Cancer, 2002
- In each of these cell lines, the genes examined included ERCC1, XPA, XPB, XPD, XPG  [ **ERCC5** ] , CSB  [ **ERCC6** ]  and p53.  Ref: 10738106 Cancer Lett, 2000
- P< ; 10 11 while XPA, XPB, XPC, XPG  [ **ERCC5** ] , XPFand CSB  [ **ERCC6** ]  mRNAs were moderately correlated r=0.2 0.7.  Ref: 11056291 Mutat Res, 2000
- XPG  [ **ERCC5** ]  mRNA from XPA cells and normal control cells was not markedly transcribed by HuIFN beta treatment compared to that from CSB  [ **ERCC6** ]  cells.  Ref: 9864391 Int J Mol Med, 1999
- We observed a 12.2 and 12.5% decrease in the baseline expression levels of XPG  [ **ERCC5** ]   **ERCC5**  and CSB  [ **ERCC6** ]   **ERCC6** , respectively, in cases compared with controls.  Ref: 10910954 Carcinogenesis, 2000
- Additionally we have examined interactions between TFIIH components, the human NER protein XPG  [ **ERCC5** ] , and the CSB  [ **ERCC6** ]  protein which is implicated in Cockayne syndrome CS.  Ref: 8652557 Biochemistry, 1996
- In multivariate logistic regression analysis, low expression of ERCC1, XPB ERCC3, XPG  [ **ERCC5** ]   **ERCC5** , and CSB  [ **ERCC6** ]   **ERCC6**  was associated with a statistically significant increased risk for SCCHN.  Ref: 11900225 Cancer, 2002
- mRNA levels of XPA, XPB, XPC, XPD, XPF, XPG  [ **ERCC5** ] , CSB  [ **ERCC6** ]  and ERCC1 in primary lymphocytes from 33 individuals were quantified by dot blots and normalized to beta actin.  Ref: 11056291 Mutat Res, 2000
- We examined the modulation of XPG  [ **ERCC5** ]  gene expression by HuIFN beta in UV sensitive cells from Cockayne syndrome complementation B CSB  [ **ERCC6** ] , xeroderma pigmentosum complementation A XPA and normal control cells.  Ref: 9864391 Int J Mol Med, 1999
- Clinically observed similarities between COFS syndrome and CS have been followed by discoveries of cases of COFS syndrome that are associated with mutations in the XPG  [ **ERCC5** ]  and CSB  [ **ERCC6** ]  genes.  Ref: 11443545 Am J Hum Genet, 2001
- these can be further subdivided into mutants which are defective in nucleotide excision repair alone UV5 ERCC2, UV24 ERCC3, UV135  **ERCC5**  and UV61  **ERCC6**  compared to those which have an associated defect in the distinct but overlapping pathway for the repair of DNA crosslinks UV20 ERCC1 and UV41 ERCC4.  Ref: 9339946 Radiat Res, 1997
- Our results suggest that individuals whose expression levels of XPG  [ **ERCC5** ]   **ERCC5**  and CSB  [ **ERCC6** ]   **ERCC6**  are reduced may be at higher risk of lung cancer.  Ref: 10910954 Carcinogenesis, 2000
- Loss of heterozygosity LOH analysis for the XP, XPA, XPB, XPC, XPD, XPE, XPF, XPG  [ **ERCC5** ]  and the transcription coupled repair factor, Cockayne syndrome B CSB  [ **ERCC6** ]  revealed that NER factors were abnormal in 62.1 % of ovarian tumors 18 2 6.7% of colon 2 12 and 22.2% lung 2 9 carcinomas.  Ref: 11689286 Cancer Lett, 2001
- Loss of heterozygosity LOH analysis, using the microdissected tissues, for the XPA, XPB, XPC, XPD, XPE, XPF, XPG  [ **ERCC5** ]  and the transcription coupled repair factor, Cockayne syndrome B CSB  [ **ERCC6** ]  revealed that NER factors were abnormal in 30.0% 3 10 cases of oral squamous cell carcinomas.  Ref: 11496330 Oncol Rep< MedlineTA>Oncol Rep, 2001
- Defects in nucleotide excision repair NER as defined by the UV sensitivity of xeroderma pigmentosum XP, Cockayne syndrome CS and trichothiodystrophy TTD patients has lead to the identification of most of the genes involved XPA through XPG  [ **ERCC5** ] , CSA and CSB  [ **ERCC6** ] .  Ref: 11104904 Mutat Res, 2001
- Transcription coupled repair of 8 oxoguanine requirement for XPG  [ **ERCC5** ] , TFIIH, and CSB  [ **ERCC6** ]  and implications for Cockayne syndrome.Analysis of transcription coupled repair of oxidative lesions here reveals strand specific removal of 8 oxo guanine and thymine glycol both in normal human cells and xeroderma pigmentosum cells defective in nucleotide excision repair.  Ref: 10786832 Cell, 2000
